# Retraction: Hsia, T.-C., et al. Advanced Glycation End-Products Enhance Lung Cancer Cell Invasion and Migration. *Int. J. Mol. Sci.* 2016, *17*, 1289, doi:10.3390/ijms17081289

**DOI:** 10.3390/ijms18081642

**Published:** 2017-07-28

**Authors:** International Journal of Molecular Sciences Editorial Office

**Affiliations:** MDPI AG, St. Alban-Anlage 66, 4052 Basel, Switzerland

In article [1], several Western blot bands in Figures 1–4 have been questioned due to similarity. The authors’ usual protocol is to photograph the final Western blot bands save them as TIF files. Unfortunately, the authors did not keep TIF files for all of the questioned bands. Thus, by mutual agreement between the authors and the journal, the article will be retracted. The authors apologize for any inconvenience caused, and intend to carry out the reported experiments again and submit it as a new article.

The article will be marked as retracted. The *International Journal of Molecular Sciences* is a member of COPE and takes research integrity, including the correct reporting of figures, very seriously.
